# Two-photon fluorescent probes for detecting the viscosity of lipid droplets and its application in living cells†

**DOI:** 10.1039/d0ra09683k

**Published:** 2021-02-22

**Authors:** Huiying Chen, Jianzhi Zhao, Junzhi Lin, Baoli Dong, Hui Li, Bing Geng, Mei Yan

**Affiliations:** School of Chemistry and Chemical Engineering, University of Jinan Jinan 250022 China chm_gengb@ujn.edu.cn chm_yanm@ujn.edu.cn; Institute of Fluorescent Probes for Biological Imaging, School of Chemistry and Chemical Engineering, University of Jinan Jinan 250022 China; Shandong Provincial Key Laboratory of Fluorine Chemistry and Chemical Materials, University of Jinan Jinan 250022 China

## Abstract

Lipid droplets (LDs) are storage organelles at the centre of lipid and energy homeostasis, which act as vital hubs of cellular metabolism and the key to maintaining lipid and energy homeostasis. We synthesized a new two-photon fluorescent probe (CIV) that could detect the viscosity of lipid droplets. The probe is constructed *via* the typical ICT system of D–π–A using carbazole as the donor and imidazole as the acceptor. With the increase in viscosity from PBS to 99% glycerol, the fluorescence intensity of CIV increased by 13-fold, showing sensitivity and specificity towards viscosity. In addition, CIV showed low toxicity and excellent biocompatibility in cytotoxicity tests, and was successfully used for living cell LD imaging. Taken together, the results widen the way for the development of novel fluorescent probe-based the visualization LDs and detection in solutions, physiology and pathology.

## Abstract 2

A novel two-photon fluorescence probe (CIV) can detect the viscosity and locate lipid droplets in living cells.

## Graphical abstract

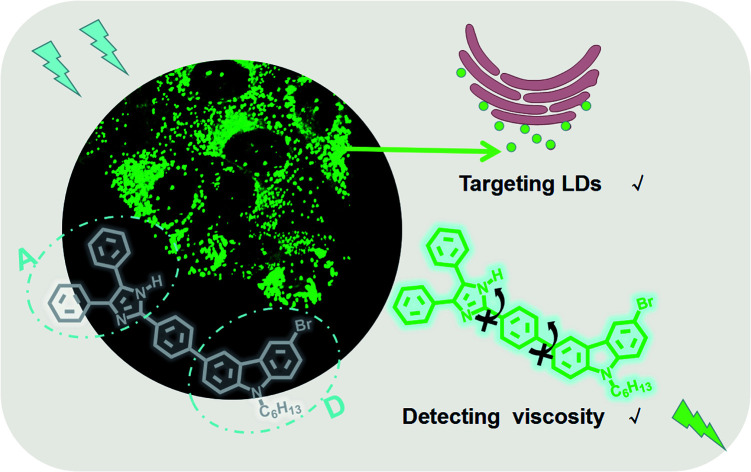

## Introduction

Lipid droplets (LDs) are organelles that store lipids to produce energy and maintain cell homeostasis.^1–3^ They are ubiquitous and highly dynamic in cells, which can associate with most other organelles through membranes and participate in numerous important physiological processes (such as cell proliferation, apoptosis and migration).^4,5^ Abnormal viscosity in LDs can destroy the interactions between different organelles in the organism and cellular metabolism, and even cause potential lipid toxicity, which can lead to diseases such as Alzheimer's disease, high blood pressure, atherosclerosis and hyperlipidemia.^6–9^ Therefore, it has become apparent to explore a fast, effective and simple method to detect the viscosity in LDs. The fluorescence imaging technology has become the most commonly used non-invasive method to visualize cell components due to its excellent spatial–temporal resolution, good optical performance and real-time imaging.^10–16^ Thence, fluorescent probe imaging is an excellent method to detect the viscosity of LDs.

However, most of the fluorescent probes in the past are one-photon (OP) properties. OP imaging is excited by a short and high-energy photon, but its penetration depth is shallow, which causes a series of problems, including photobleaching, photodamage and low fluorescence.^17–19^ Therefore, the applications of OP probes are limited in biological samples and organisms. Fortunately, the two-photon (TP) imaging uses two lower-energy photons as the excitation source. The long excitation wavelength gives it numerous advantages over OP imaging, such as lower background fluorescence, causes less damage to biological samples, less photobleaching, less heating effects, better 3D spatial positioning and deeper penetration depth.^20–23^ Because of these advantages, TP imaging is becoming increasingly popular among biologists. To date, a variety of TP fluorescence probes have been synthesized and their applications in biological imaging have been proved.^24–28^ Kim *et al.*^29^ designed a novel TP fluorescent probe using azulene as the fluorophore, which was used for the detection of peroxynitrite and showed excellent photostability and low toxicity. Yao^30^ has synthesized a new TP small molecule enzyme probe based on photocatalytic properties to amplify fluorescent signals, which can directly evaluate the functions of enzyme activity in complex pathological environments, and has good photophysical properties and deep Penetration. Peng^5,31^*et al.* reported several TP fluorescent probes using carbazole as the donor to detect viscosity. However, the response folds of the as-prepared probes are less than 10-fold and the fluorescence quantum yields are less than 10%. Niu^32,33^*et al.* had prepared several TP fluorescent probes using phenanthrenequinone imidazole as the fluorophore core. Niu's TP probe could only locate lipid droplets and could not detect viscosity, reactive oxygen species (ROS) and ions *etc.*

**Scheme 1:**
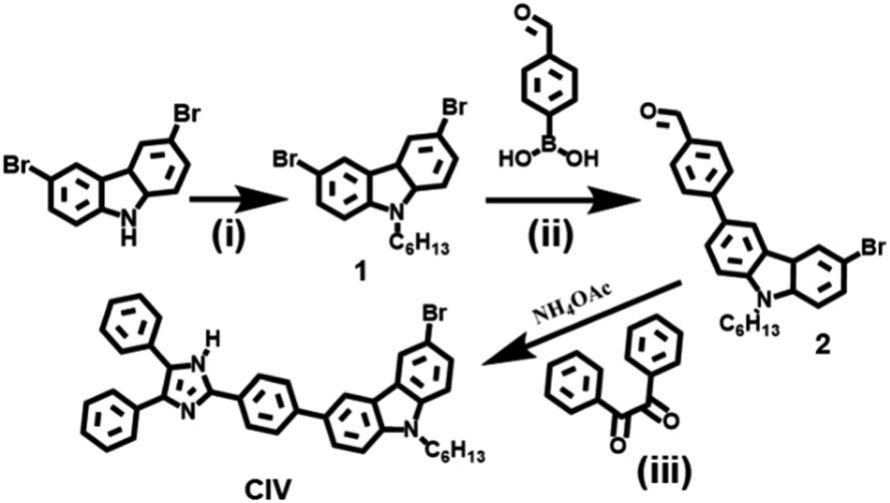
Synthetic route of CIV. (i) NaOH, bromohexane, 55 °C, 12 h; (ii) 4-formylphenylboronic acid, K_2_CO_3_, tetrakis(triphenylphosphine) platinum, N_2_, 55 °C, 12 h; (iii) 1,2-diketone, ammonium acetate, l-proline, 60 °C, 10 h.

In this study, a novel TP fluorescence probe (CIV) with higher viscosity response folds and fluorescence quantum yields in solution or in LDs was synthesized (Scheme 1). The probe conforms to the design concept of traditional fluorescent probes for viscosity detection,^34^ and the typical ICT system (D–π–A) was constructed with carbazole and imidazole in the fluorescent probe CIV, simultaneously. The carbazole used as a strong electron-donating group and imidazole as electron-accepting group are connected by a benzene ring. In the case of low viscosity, the single bond between the benzene ring and carbazole rotates freely, and the whole probe is not coplanar, which hardly shows fluorescence. The fluorescence intensity increased significantly due to the suppression of the rotor free rotation and enhancement of the conjugation length in the system with relatively high viscosity. In addition, imidazole with the asymmetric five-membered heterocyclic ring endows the TP probe with large conjugated system, which could increase the fluorescence quantum yield, stokes shift and response multiples. The hexyl group on carbazole can increase the lipid compatibility of the probe. Based on the above-mentioned strategy, we predict that fluorescent probe CIV can be used to detect the viscosity of lipid droplets and perform TP imaging.

## Experimental

### Materials

All reagents and chemicals used in this article are of analytical grade unless otherwise stated. All of the chemicals for reaction were used directly from commercial resources and without any further purification. Sodium hydroxide and potassium carbonate were purchased from Damao Chemical Reagent Factory. All other chemicals used are purchased from Aladdin and all reagents used are from Fuyu Chemical Reagent Factory. Doubly distilled water was used throughout all experiments.

### Synthesis of compound 1

First, 3, 6-dibromocarbazole (3.25 g, 10 mmol) and NaOH (0.8 g, 10 mmol) were dissolved in 15 mL of the mixed solvent (v_THF_ : v_H_2_O_ = 1 : 3), and stirred at room temperature for 1.5 h. Then, bromohexane (9.90 g, 60 mmol) was added and the reaction mixture was heated at 55 °C for 12 h. The crude product was purified *via* silica gel column chromatography (petroleum ether/ethyl acetate, 30 : 1) to obtain compound 1 (white product; 70%)

### Synthesis of compound 2

A mixture of compound 1 (409 mg, 1 mmol), 4-formylphenylboronic acid (180 mg, 1.2 mmol), K_2_CO_3_ (447 mg, 0.03 mmol) and tetrakis(triphenylphosphine) platinum in THF (10 mL) was heated to 55 °C and stirred for 12 h under nitrogen. After the reaction, the solvent was removed under a reduced pressure. The crude product was purified *via* silica gel column chromatography (petroleum ether/ethyl acetate, 30 : 1) to obtain compound 2 (yellow product; 67%)

### Synthesis of CIV

Compound 2 (217.5 mg, 0.5 mmol), 1,2-diketone (105 mg, 0.5 mmol) and ammonium acetate (77 mg, 1 mmol) were taken in the presence of l-proline in methanol (34.5 mg, 15 mol%) at room temperature, and stirred until dissolved. The whole mixture reacted at 60 °C for 10 h. After completion of the reaction, the volume of the solvent was reduced.^35^ The crude product was purified by silica gel column chromatography (dichloromethane/methanol, 30 : 1) to obtain compound CIV (yellow product; 74%).

The detailed ^1^H NMR, ^13^C NMR and HRMS spectra of CIV are shown in the Fig. S1, S2 and S3.†

## Results and discussion

### Fluorescence mechanism

**Scheme 2:**
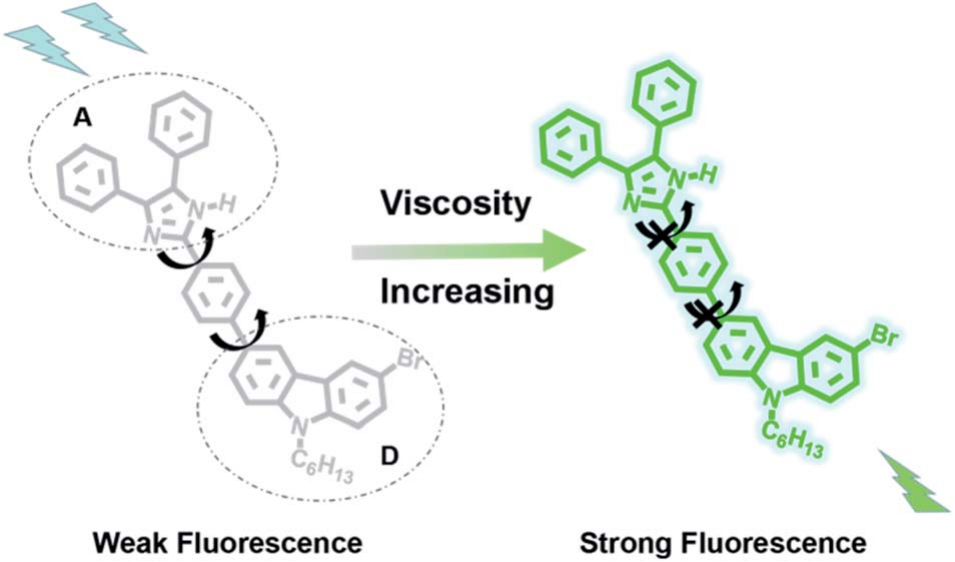
The mechanism of the probe CIV.

Carbazole has become an excellent fluorescent probe matrix due to its special rigid planar structure and large conjugated system, which endow it with high thermal and optical stability and good photophysical properties.^36^ Therefore, we used carbazole (donor, D) as the source, connected imidazole (acceptor, A) through the benzene ring at 3 positions of carbazole, and constructed a typical ICT system of D–π–A in CIV (Scheme 2). In the case of low viscosity, the free rotation of the single bond between the benzene ring and carbazole makes the whole probe non-coplanar, so the fluorescence of CIV is very weak. The fluorescence intensity increased significantly due to the inhibition of the free rotation of the single bond in the system with relatively high viscosity such as glycerol. The hexyl group at position 9 of carbazole can increase the lipophilicity of molecules, which makes it coincide with lipid droplets better and numerous carbazole derivatives have been designed and confirmed as TP probes. Based on the above-mentioned strategy, we predict that fluorescent probe CIV can be used to detect the viscosity of lipid droplets and perform TP imaging.

### Absorption and emission spectroscopy

**Fig. 1:**
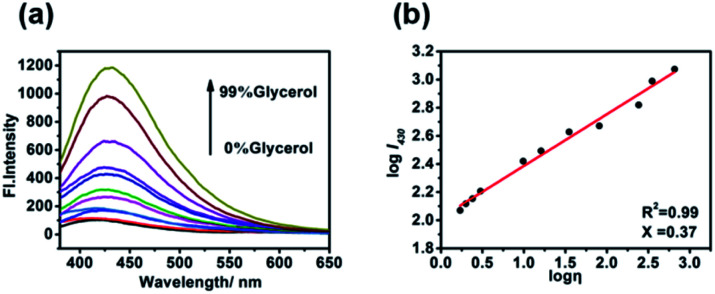
(a) The fluorescence emission spectra of 10 μM·of CIV with *λ*_ex_ = 340 nm in the varied viscosity media of the PBS/glycerol (v/v) mixtures; (b) linear relationship of log *I*_430_ and log *η*.

For the probe CIV, first, we studied its optical properties. The result of the UV absorption spectra in pure PBS and pure glycerol shows an increase in the absorption intensity of CIV in glycerol compared to that in PBS under the same concentration (Fig. S4†). Unexpectedly, during the measurement of the fluorescence spectrum, the fluorescence emission intensity of CIV increased by 13-fold with the volume ratio of glycerol in the mixed solution increasing from PBS to 99% glycerol (Fig. 1a). This indicates that the fluorescent probe CIV has a good response towards viscosity. To further explore the probe, we studied the relationship between the wavelength and viscosity and obtained the correlation coefficient of the linear relationship that reached 0.99 between log *I*_430_ and log *η via* the Förster–Hoffmann equation^37^ (Fig. 1b). This indicates a good linear relationship, and the CIV probe is suitable for the quantitative determination of viscosity. The fluorescence quantum yields (Fig. S5†) and the molar extinction coefficients (Fig. S6†) of CIV in glycerol were higher than in PBS, which is consistent with the spectrum test. The tests of the TP absorption cross-section (Fig. S7a†) and TP fluorescence curves (Fig. S7b†) in PBS and glycerol showed that CIV was a TP fluorescent probe.

**Fig. 2:**
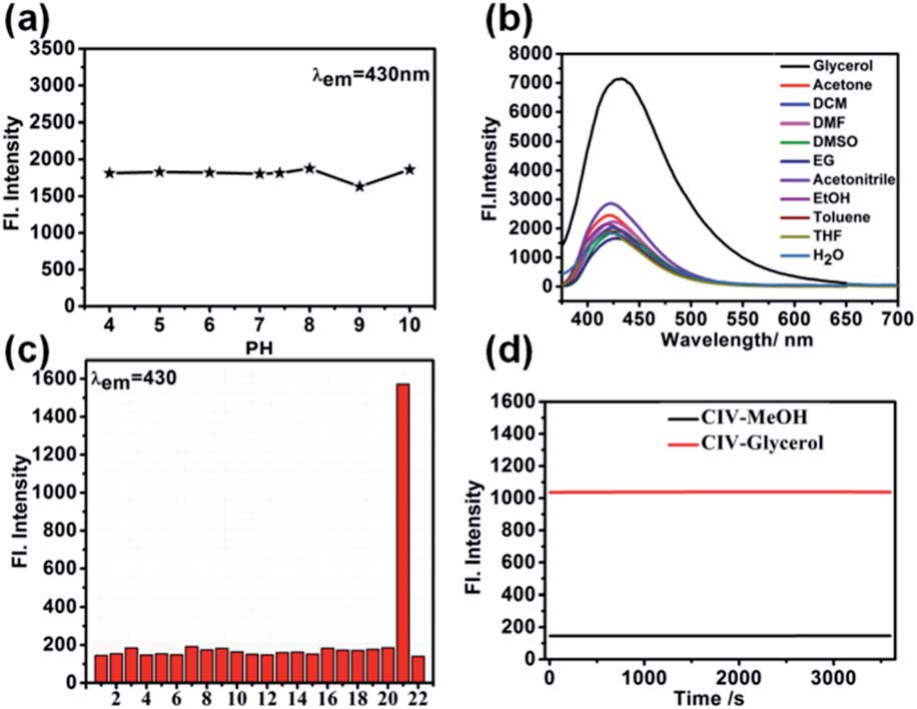
(a) The fluorescence emission spectra of CIV (10 μM) with *λ*_em_ = 430 nm in the varied pH(PBS, pH = 7.4); (b) fluorescence emission of CIV (10 μM) in various solvents with different polarities; (c) fluorescence intensity of CIV at *λ*_em_ = 430 nm in PBS buffer (pH = 7.4, 10 μM) (1) Al^3+^, (2) Ba^2+^, (3) Ca^2+^, (4) CNS^−^, (5) Co^2+^, (6) Cu^2+^, (7) Cys, (8) acetic acid, (9) F^−^, (10) Fe^2+^, (11) Fe^3+^, (12) GSH, (13) H_2_O_2_, (14) Mg^2+^, (15) Ni^2+^, (16) NO, (17) NO_3_^−^, (18) S_2_O_3_^2−^, (19) Sn^2+^, (20) SO_3_^2−^, (21) glycerol, (22) PBS; (d) the photostability of the probe CIV in glycerol and PBS with the continuous irradiation by laser light for 60 min.

The intracellular environment is complex, and probe CIV may respond to different environments or heterogeneity, which affects its response towards viscosity. Therefore, we simulated the potential performance variations of the probe in different pH, polar environments and in the presence of cations, anions, amino acids, and active oxygen by fluorescence spectroscopy. First, the fluorescence changes of CIV in buffer solutions with different pH values of 4–10 were tested, and the results showed that it was stable without significant changes (Fig. 2a and S8†). Second, we examined the impact of different polar solvents, and the final result showed that it has little effects on the probe (Fig. 2b). The test results in the presence of numerous ions showed that these heterogeneities had negligible effects on the probe compared to viscosity (Fig. 2c). Finally, CIV showed excellent light stability both in PBS and glycerol after irradiating the CIV solution and solids by a certain light source (Fig. 2d and S9†).

### Fluorescence imaging of the CIV probe in living cells

**Fig. 3:**
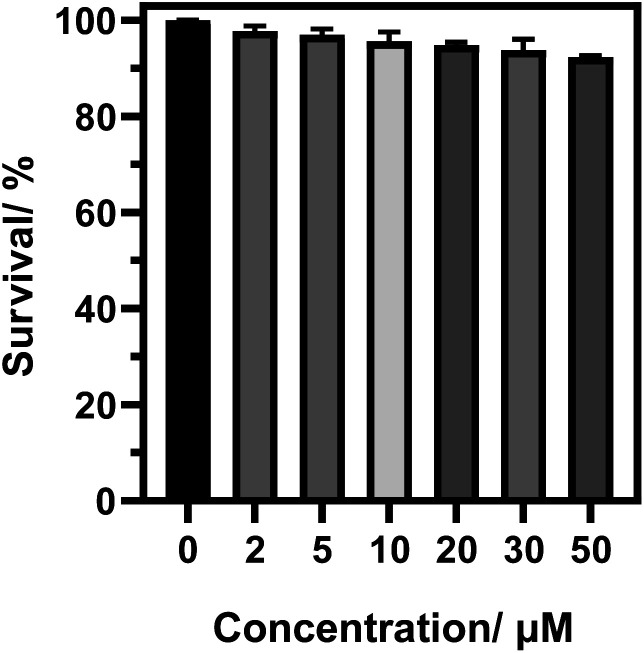
The cytotoxicity of the probe CIV in HeLa cells.

Cytotoxicity test is one of the criteria for testing whether the fluorescent probe CIV can be used for living cell imaging. Therefore, we tested the cytotoxicity of the probe by the standard MTT analysis. As shown in Fig. 3, when the living cells were incubated for 24 h in CIV concentration range of 0–50 μM, the cell survival rate was still above 90%, which indicates that CIV has great biocompatibility and low cytotoxicity.

**Fig. 4:**
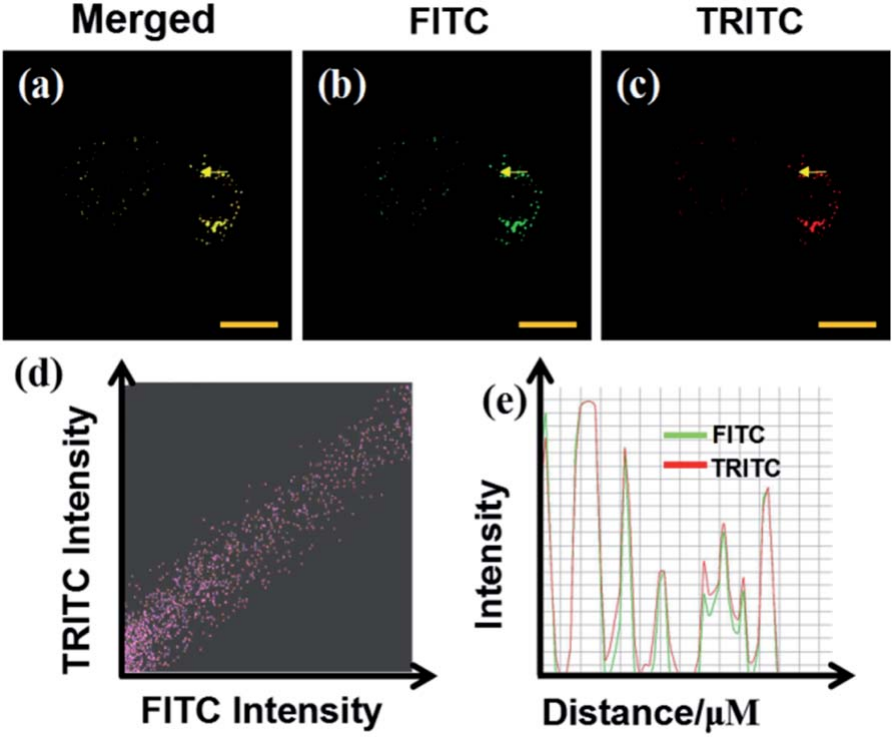
Co-localization fluorescence images of the probe CIV in HeLa cells; (a) the merged image of (b) and (c); (b) CIV (10 μM) stain, *λ*_ex_ = 405 nm, collected at 500–550 nm; (c) Nile Red stain, *λ*_ex_ = 561 nm, collected at 570–620 nm; (d) intensity scatter plots of the probe CIV across the HeLa cells in the red and green channels. (e) Intensity profile of the two channels.

Then, we verified the specific targeting ability of the fluorescent probe CIV towards lipid droplets. Co-localization experiments were performed with commercial dye Nile Red.^38^ As shown in Fig. 4, the cells were observed under a confocal microscope. The probe showed green fluorescence in the FITC channel (Fig. 4b) and Nile Red showed red fluorescence in the TRICT channel (Fig. 4c). The experiments that can exclude the interference of CIV in the FRICT channel and Nile red in the FRIT channel at 405 nm excitation had been finished (Fig. S10†). The merged image (Fig. 4a) showed that the two channels overlap very well, and the high overlap coefficient of 0.928 is obtained.

**Fig. 5:**
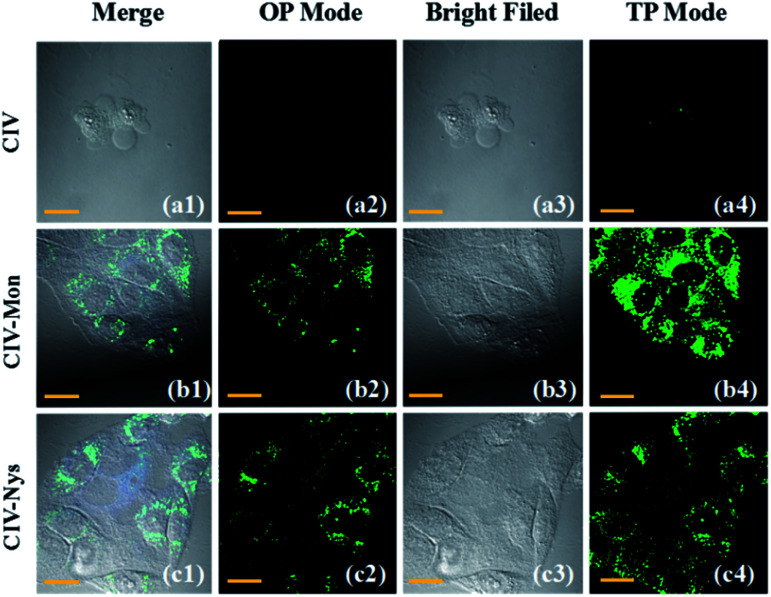
(a1–a4) Confocal fluorescence images of the HeLa cells incubated with 10 μM CIV for 30 min; (b1–b4) confocal fluorescence images of the HeLa cells incubated with 10 μM CIV + 10 μM monensin for 1 h; (c1–c4) confocal fluorescence images of the HeLa cells incubated with 10 μM CIV + 10 μM nystatin for 1 h. OP: *l*_ex_ = 405 nm; TP: *l*_ex_ = 820 nm; 500–550 nm were collected.

Monensin and Nystatin as external stimuli can induce abnormalities, including swelling or structural changes of cells, and ultimately increase intracellular viscosity, in the intracellular environment.^25,39^ Therefore, they are often used as ionophores to externally interfere with changes in the intracellular viscosity. As observed from the confocal microscope images, when the cells were incubated for 30 min only with the CIV probe, they emitted weak green fluorescence (Fig. 5a), while the cells stimulated by monensin and nystatin emitted strong fluorescence(Fig. 5b and c). Obviously, as the intracellular viscosity increases, the fluorescence intensity of the probe CIV will increase, which is consistent with the experimental results of fluorescence spectroscopy. The most important thing is that the effect of the TP imaging is clearer than that of one-photon (Fig. 5a4–c4). We confirmed again that the TP fluorescence in cells was caused by the probe CIV rather than intracellular riboflavin, flavin *etc.* (Fig. S11†). This shows that CIV has TP performance, which is less damaging to cells and superior penetration depth.

## Conclusions

In summary, we synthesized a novel TP fluorescent probe (CIV) for detecting the viscosity of lipid droplets. CIV shows excellent sensitivity towards viscosity and the fluorescence emission intensity increased by 13-fold with the increase in the volume ratio of glycerol in the mixed solution, from PBS to 99% glycerol. In addition, CIV also has low toxicity, excellent biocompatibility and good TP performance. The probe can accurately target lipid droplets in living cells and detect viscosity. The TP fluorescent probe synthesized by us may be a potential candidate for viscosity detection in solutions and complex biological systems.

## Conflicts of interest

There are no conflicts of interest to declare.

## Supplementary Material

RA-011-D0RA09683K-s001
