# Supplementary material for: Two-photon fluorescent probes for detecting the viscosity of lipid droplets and its application in living cells
Source: RSC Adv. 2021 Feb 22;11(14):8250–4. doi: 10.1039/d0ra09683k (PMC8695079; doi:10.1039/d0ra09683k)
Supplement: RA-011-D0RA09683K-s001 [file RA-011-D0RA09683K-s001.pdf]

Electronic Supplementary Material (ESI) for RSC Advances.
This journal is © The Royal Society of Chemistry 2021

Supporting Information

Two photon fluorescent probe for detecting lipid droplets viscosity and its application in living cells

Huiying Chen‡†, Jianzhi Zhao‡†, Junzhi Lin‡†, Baoli Dong‡†, Hui Li‡ , Bing Geng*‡ , and Mei Yan*‡

* Corresponding Authors

A Shandong Provincial Key Laboratory of Fluorine Chemistry and Chemical Materials, University of Jinan, Jinan 250022, China.

‡ School of chemistry and chemical engineering, University of Jinan, Jinan 250022, China

† Institute of Fluorescent Probes for Biological Imaging, School of Chemistry and Chemical Engineering, School of Materials Science and Engineering, University of Jinan, Jinan, Shandong 250022, P. R. China.

Contents

Characterization 3

Synthesis 4

Spectral test 5

Cytotoxicity assay 6

Fluorescence imaging in cells 7

Table S1 8

Figure S1 9

Figure S2 10

Figure S3 11

Figure S4 12

Figure S5 12

Figure S6 13

Figure S7 13

Figure S8 14

Figure S9 14

Figure S10 15

Figure S11 15

Characterization

^{1}H and ^{13}C NMR spectra were measured on an Avance III 400MHz spectrometer (Bruck Byerspin, Switzerland) in DMSO-d_6. High-resolution mass spectra (HRMS) were measured on an Apex-Ultra Bruke instrument. The UV-visible-near-IR absorption spectra of dilute solutions were recorded on a U2910 spectrophotometer using a quartz cuvette having 1 cm path length. One-photon fluorescence spectra of dilute solutions were obtained on a HITACH F-2700 spectrofluorimeter equipped with a 450-W Xe lamp. Confocal fluorescence images were obtained with a Nikon A1R confocal laser scanning microscope. To acquire the fluorescence images, TLC analysis was performed on silica gel plates and column chromatography was conducted over silica gel (mesh 200-300), both of which were obtained from the Qingdao Ocean Chemicals. The measurements were carried out with a NDJ-8S rotational viscometer and each viscosity value was recorded. The buffer solution of **Figure 2 (a)** was phosphate buffer saline solution (PBS, pH=7.4) and **Figure S8** was britton-robinson buffer solution (BR).

Synthesis

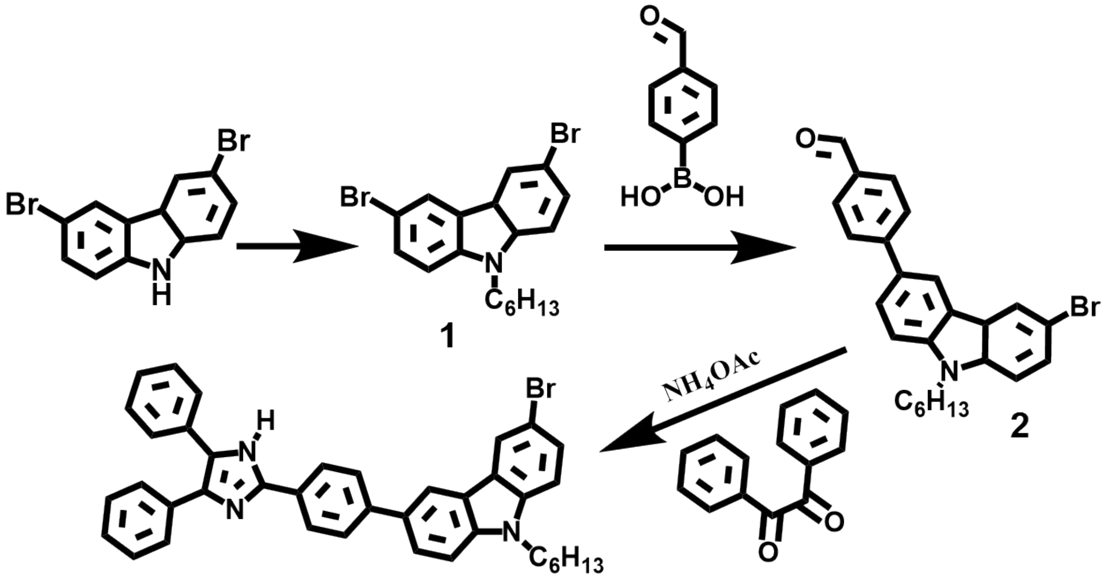

Scheme S1 Synthetic route of **CIV.**

Synthesis of compound 1

First, 3, 6-Dibromocarbazole (3.25 g, 10 mmol) and NaOH (0.8 g, 10 mmol) were dissolved in 15 mL of the mixture (V_{THF} : V_{H2O} = 1:3), and stirred at room temperature for 1.5 h. Then bromohexane (9.90g, 60mmol) was added and the reaction mixture was heated at 55°C for 12 h. The crude product was purified by silica gel column chromatography (petroleum ether/ethyl acetate, 30:1) to obtain compound 1 (white product; 70%)

Synthesis of compound 2

A mixture of compound 1 (409 mg, 1 mmol), 4-formylphenylboronic acid (180 mg, 1.2 mmol), K_2CO_3 (447 mg, 0.03 mmol) and tetrakis(triphenylphosphine) platinum in THF (10 ml) was heated to 55°C and stirred for 12 h under nitrogen. After the

reaction, the solvent was removed under reduced pressure. The crude product was purified by silica gel column chromatography (petroleum ether/ethyl acetate, 30:1) to obtain compound 2 (yellow product; 67%)

Synthesis of CIV

Compound 2 (217.5mg, 0.5mmol), 1,2-diketone (105mg, 0.5mmol) and ammonium acetate (77mg, 1mmol) were taken in presence of L-proline in methanol (34.5mg, 15 mol %) at room temperature, and stir until dissolved. The whole mixture reacted at 60 °C for 10 hours. After completion of the reaction, volume of the solvent was reduced. The crude product was purified by silica gel column chromatography (dichloromethane/methanol, 30:1) to obtain compound **CIV** (yellow product; 74%). ^{1}H NMR (400 MHz, DMSO) δ 12.76 (s, 1H), 8.78 (s, 1H), 8.66 – 8.62 (m, 1H), 8.23,8.21 (d, J = 8.3 Hz, 2H), 7.99-7.91 (dd, J = 21.7, 8.5 Hz, 4H), 7.75,7.72 (d, J = 8.5 Hz, 1H), 7.60-7.54 (dd, J = 17.5, 7.3 Hz, 5H), 7.49-7.45 (t, J = 7.5 Hz, 2H), 7.41,7.40 (d, J = 7.3 Hz, 1H), 7.35-7.31 (t, J = 7.6 Hz, 2H), 7.26-7.23 (t, J = 7.4 Hz, 1H), 4.50-4.47 (m, 2H), 1.86-1.82 (d, J = 6.2 Hz, 2H), 1.23 (s, 6H), 0.85-0.82 (t, J = 7.1 Hz, 3H). ^{13}C NMR (400 MHz, DMSO) δ 167.09, 154.40, 153.35, 148.91, 135.79, 135.62, 132.11, 129.80, 126.22, 125.82, 125.58, 125.51, 124.55, 124.47, 120.96, 119.37, 118.60, 116.95, 115.03, 112.71, 110.05, 105.47, 53.40, 25.23, 24.94, 22.73, 21.87, 14.55. HRMS (ESI): calcd. for $C_{39}H_{34}BrN_3$,[M+H]+, m/z, 624.2014, found: 624.2020.

Spectral test

The solvents were obtained by mixing a PBS-glycerol system in different

proportions. Measurements were carried out with a NDJ-8 rotational viscometer, and each viscosity value was recorded. The stock solutions of the probe **CIV** (5 mM) was prepared in dimethyl sulfoxide (DMSO). In the test solution, take 2 mL of different ratios of glycerol and methanol (glycerol: PBS / V : V= 0:10, 1:9, 2:8, 3:7, 4:6, 5:5, 6:4, 7:3 , 8:2, 9:1, 10:0), then add the probe stock solution (10μM) for fluorescence scanning (excitation wavelength 340 nm, detection band 350 - 650 nm) . The test results are shown in **Figure 1a**.

A stock solution (1 mM) of sodium salt (CNS^-、F^-、NO_3^{--}、NO_2^-、$S_2O_3^{2-}$、SO_3^{2-}), chloride salt (Al^{3+}、Ba^{2+}、Ca^{2+}、Co^{2+}、Cu^{2+}、Fe^{2+}、Fe^{3+}、Mg^{2+}、Ni^{2+}、Sn^{2+}), inorganic matter (H_2O_2), Organic salt (Acetic acid) and amino acid (Cys、GSH) was prepared in deionized water.

Cytotoxicity assay

The cytotoxicities of the probe **CIV** to Hela cells was studied by standard MTT assays. 2×10^4 cells mL^{-1} cells were seeded in 96-well plates and then incubated with various concentrations of the probe (0, 2, 5, 10, 20, 30, 50 μM) for 24 h. After that, 10 μL MTT (5 $mg \cdot mL^{-1}$) was added to each well and incubated for another 4 h. Finally, the media was discharged, and 100 μL of DMSO was loaded to dissolve the formazan crystals. The plate was shaken for about 10 min, and each well was analyzed by the microplate reader and detected at the absorbance of 490 nm. The cell viability (%) = $(OD_{sample}-OD_{blank}) / (OD_{control}- OD_{blank}) \times 100$ %） .

Fluorescence imaging in cells

HeLa cells of appropriate density were cultured in 90% Dulbecco's Modified Eagle Medium (DMEM, Gibico) supplemented with 10% FBS (Hyclone) and 1% antibiotics (100 U / mL penicillin and 100 μg /mL streptomycin, Hyclone) in an atmosphere of 37 °C and 5% CO_2. The first group was added with 10 μM of fluorescent probe and cultured for 30 minutes. The second group was added with 10 μM viscosity stimulator Monensin and cultured for 30 minutes. Then the cells were incubated with the probe for additional 30 min. The third group was added 10 μM viscosity stimulating substance Nystatin and cultured for 30 minutes. Then the cells were incubated with the probe for additional 30 min. After washed with PBS for three times, the fluorescence imagings were carried out by a Nikon A1MP inverted fluorescence confocal microscope. The fluorescence emission of the probe was collected at FITC channel (500nm-550nm), the excitation wavelength of one photon imaging was 405 nm and Two photon imaging was 820 nm. Monensin and Nystatin could induce structural changes or swelling, leading to viscosity changes in the lipid droplets.

Table S1 Test viscosity in the varied of the PBS/glycerol (v/v) mixtures, Fluorescence intensity at 430nm and fluorescence quantum yield (**Φ_f**) of **CIV**.

Methanol / glycerol (v : v)	η / cP	Fl.Intensity(I_{430}) / a.u.	**CIV** - Φ_f / %
10:0	1.7	96.4	0.225
9:1	2.0	109.7	
8:2	2.4	142.5	
7:3	3	160.9	
6:4	9.8	263.2	
5:5	16.1	311.6	
4:6	35	425.3	
3:7	81	469	
2:8	242	659.7	
1:9	351	974.8	
0:10	656	1185	4.5

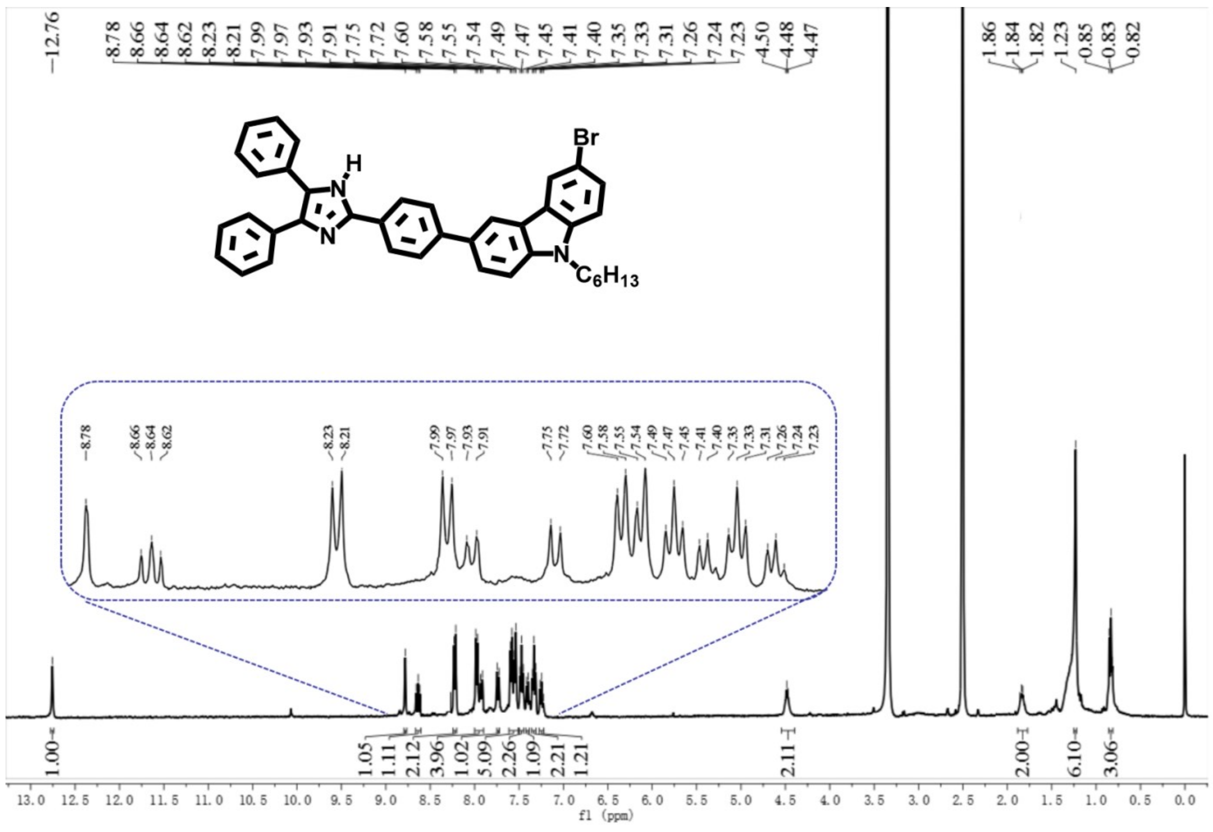

Figure S1 ^{1}H NMR spectrum of **CIV** in DMSO-d_6

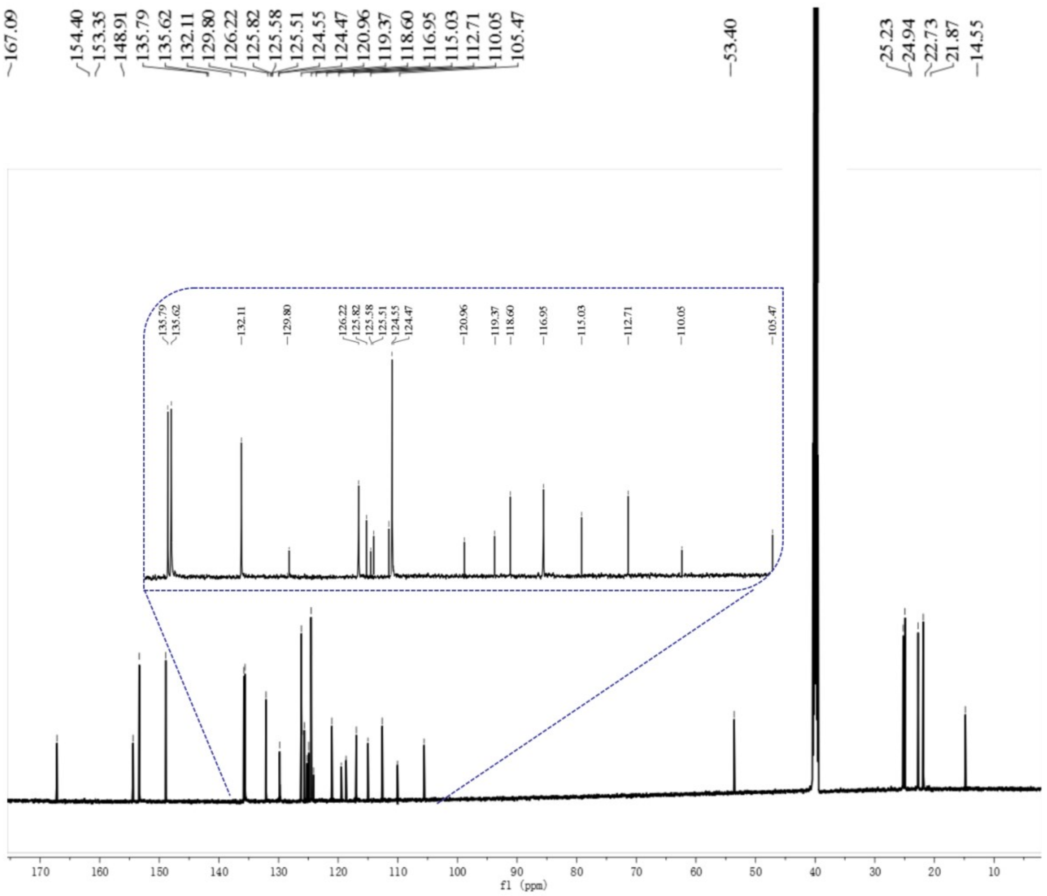

Figure S2 ^{13}C NMR spectrum of **CIV** in DMSO-d_6

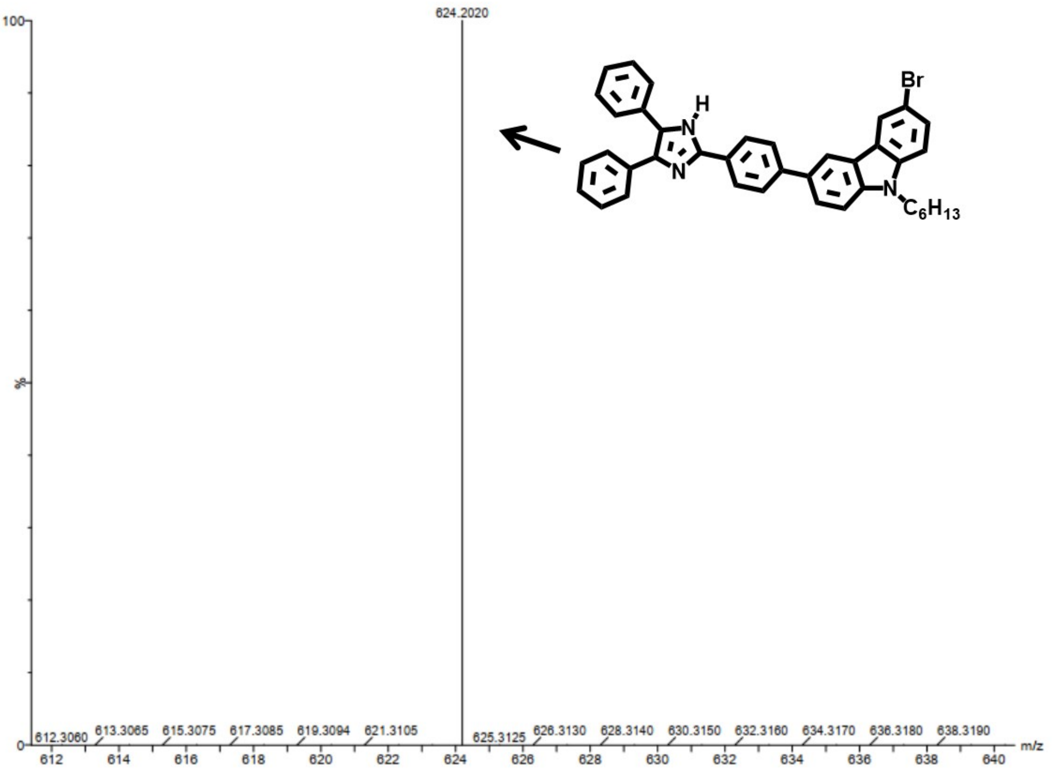

Figure S3 HRMS spectrum of **CIV**

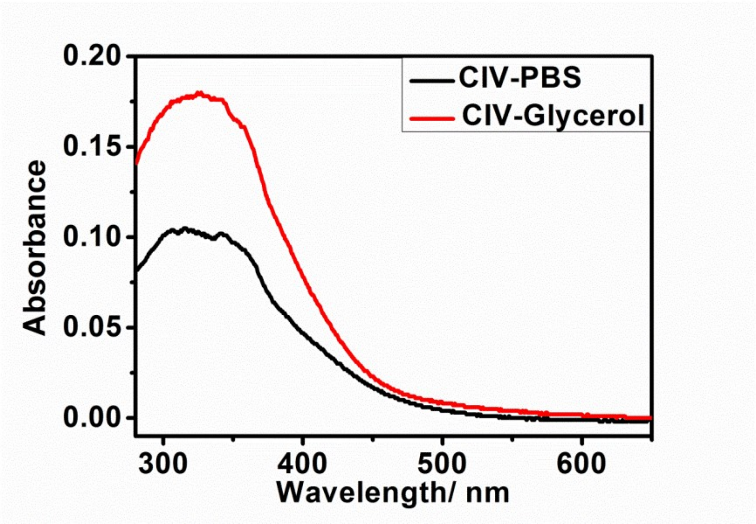

Figure S4 Absorption of probe **CIV** in PBS and glycerol

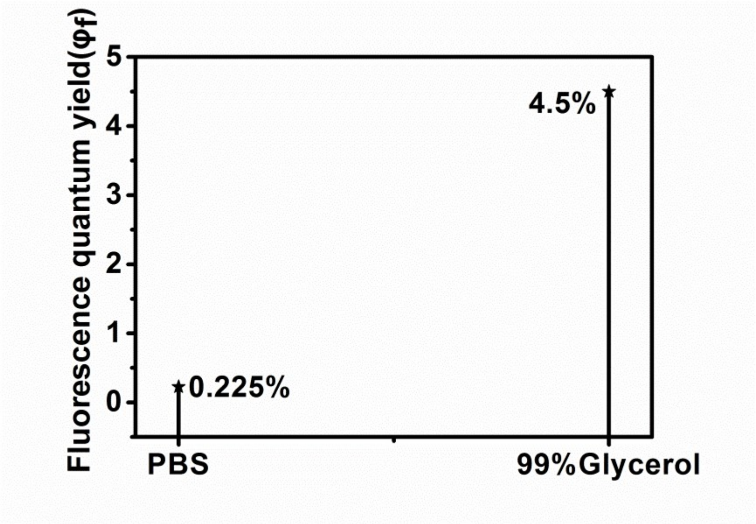

Figure S5 The fluorescence quantum yield (Φ_f) of **CIV** in PBS and 99% glycerol

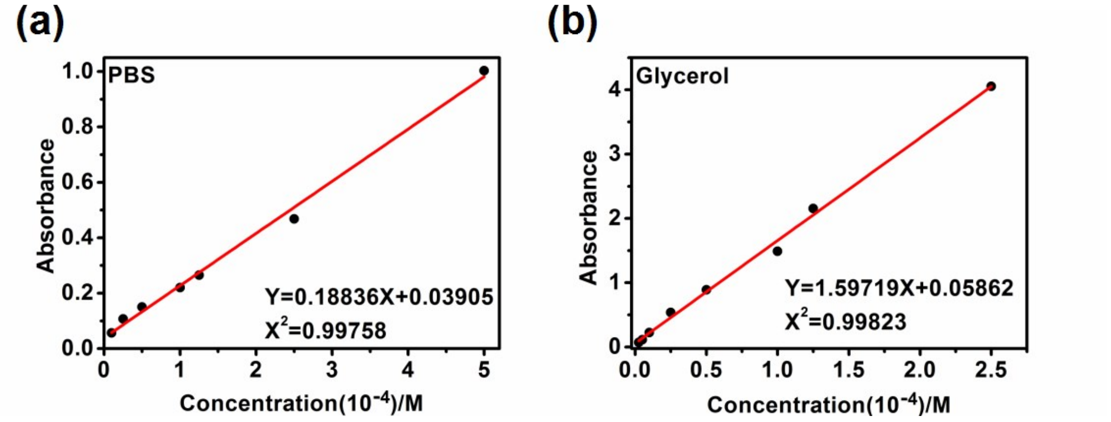

Figure S6 The molar extinction coefficient of **CIV** in PBS and 99% glycerol

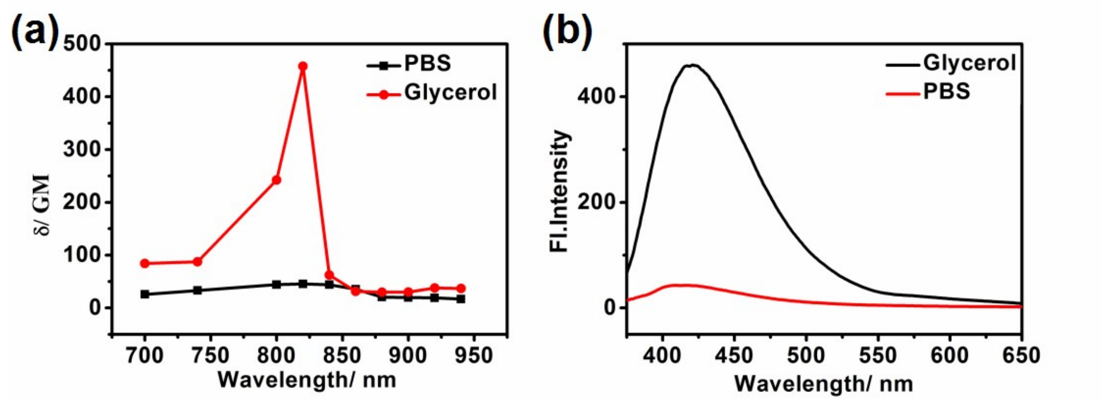

Figure S7 (a) Two-photon absorption cross-section (dF) data of **CIV** (10 μM) in PBS and 99% glycerol; (b) Two-photon fluorescence curves **CIV** (10 μM) in PBS and 99% glycerol excited by a femtosecond laser with 820 nm irradiation.

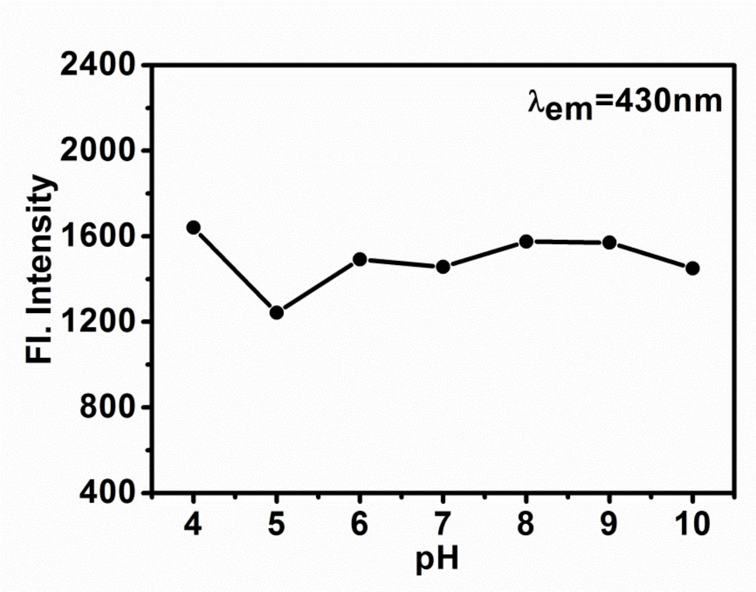

Figure S8 The fluorescence emission spectra of **CIV** (10μM) with λem = 430 nm in the varied pH (BR buffer).

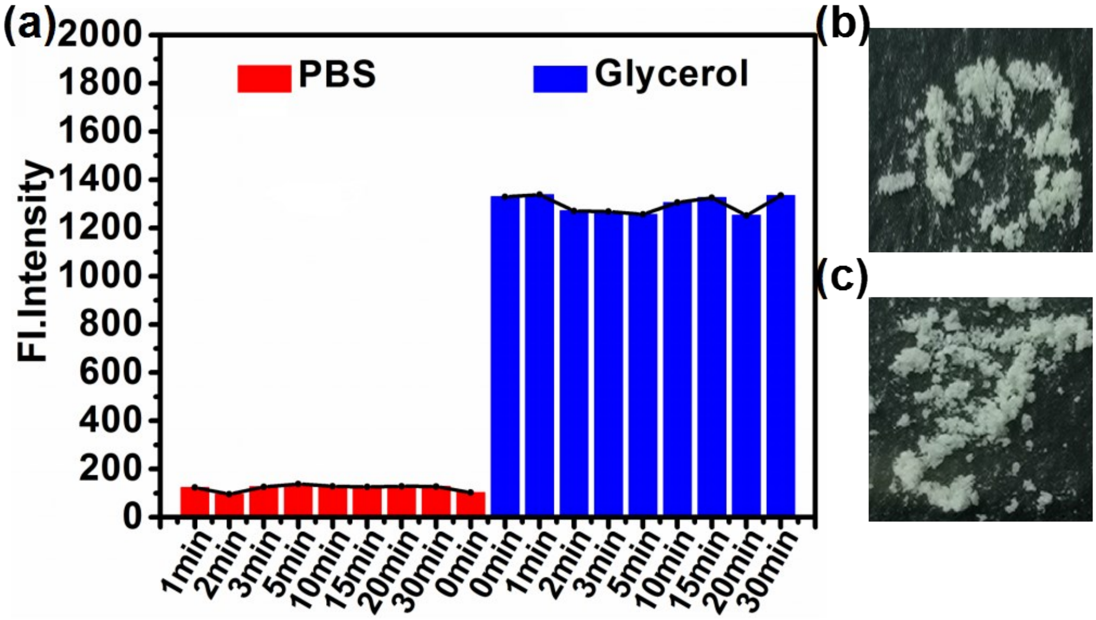

Figure S9 The photostability of the probe **CIV** on a glass slide illuminated by xenon lamp. (a) The stability of the probe after xenon lamp irradiation in PBS and glycerol; (b) Solid probe; (c) Solid probe after xenon lamp irradiation for 30 min.

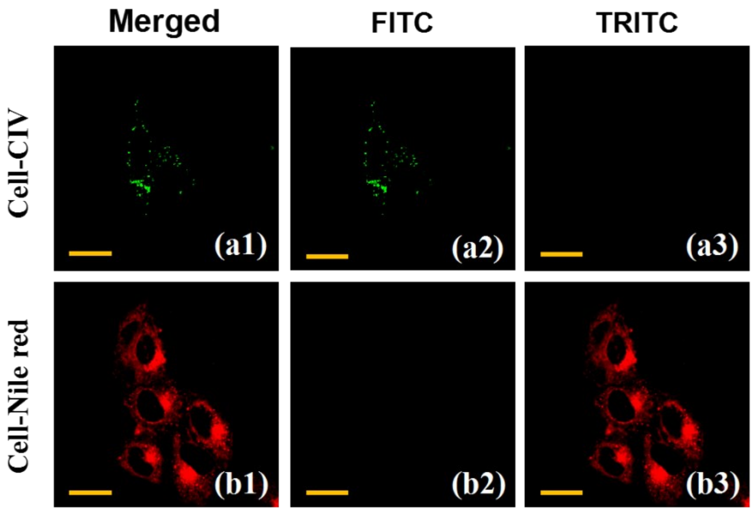

Figure S10 (a1–a3) Confocal fluorescence images of the HeLa cells incubated with 10μM **CIV** for 30 min, λex = 405 nm; collected at 500–550 nm; (b1–b3) Confocal fluorescence images of the HeLa cells incubated with 0.2μM Nile red, λex = 561 nm, collected at 570 – 620 nm.

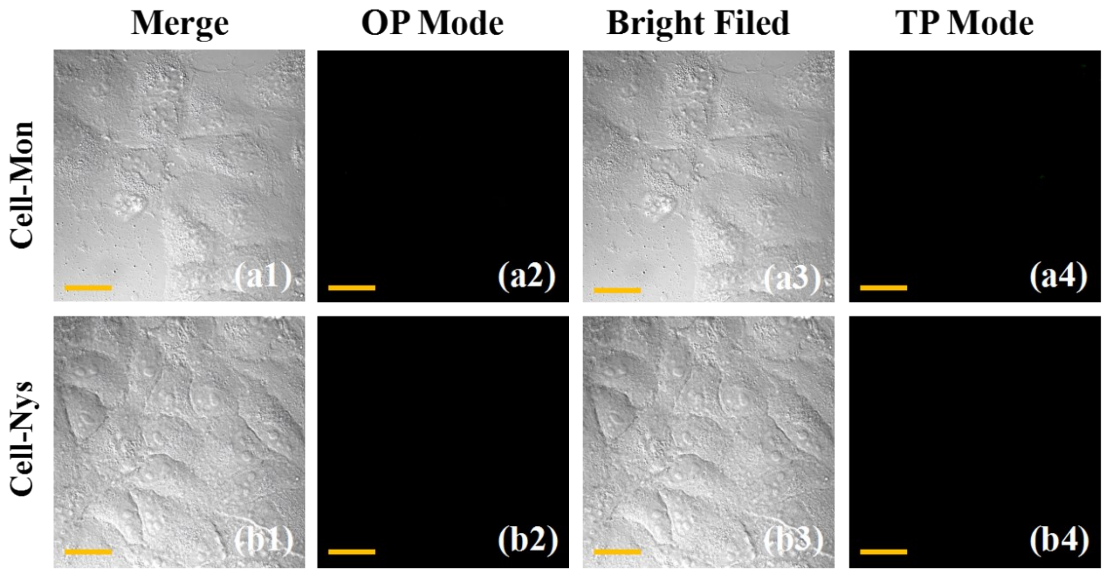

Figure S11 (a1–a4) Confocal fluorescence images of the HeLa cells incubated with 10μM monensin for 1h; (b1–b4) Confocal fluorescence images of the HeLa cells incubated with 10μM nystatin for 1 h. OP: lex = 405 nm; TP: lex = 820 nm; 500–550 nm were collected.
